# Authors’ reply

**DOI:** 10.1080/20008686.2017.1333745

**Published:** 2017-06-13

**Authors:** Kerstin Myrtennäs, Krustyu Marinov, Anders Johansson, Marcin Niemcewicz, Edvin Karlsson, Mona Byström, Mats Forsman

**Affiliations:** ^a^ Swedish Defence Research Agency, Umeå, Sweden; ^b^ Military Medical Academy, Sofia, Bulgaria; ^c^ Umeå University, Umeå, Sweden; ^d^ Military Institute of Hygiene and Epidemiology, Pulawy, Poland

We thank you for the feedback on our study about tularemia outbreaks in Bulgaria. The purpose of our study was not to make any conclusion or statement in favor of or against the use of traditional epidemiology data. We fully agree that genomic data without epidemiological and clinical data may not be enough to track the source of a pathogen. In fact, we have ourselves also emphasized this in several genomic publications on tularemia. However, the intention of the Bulgarian study was to test how bacterial strains of *Francisella tularensis* causing tularemia in wildlife and humans in the 1960s and the 1990s were genetically related.

We found that *F. tularensis* strains were remarkably similar over long time periods and noted that this finding is compatible with the ‘natural nidality of disease’ concept put forward in the 1960s which postulates that some diseases occur naturally in wildlife in certain places (nidus) over time. We also found a close genetic relationship between an isolate from a muskrat infected in 1961 in Bulgaria and an isolate from a water rat infected in 1956 in Russia. These isolates differed by two nucleotides at the whole genome level. We suggested that implantation of muskrats into Bulgaria from Russia may have introduced the disease but avoided making definitive conclusions. We think that it is scientifically sensible to admit that relying on genetic results only is not sufficient to make definite conclusions. Finally, in an attempt to respond to the request for more epidemiological information we have now updated the information in the Supplementary material.

Sincerely,

